# A New Career for Medical Men

**Published:** 1921-08-27

**Authors:** 


### A New Career for Medical Men.

### THE ROYAL AIR FORCE MEDICAL SERVICE.

The conditions of this Service and the general regulations have only been formulated during the past year. This latest Medical Service offers a career for medical men which should prove both attractive and interesting. The rates of pay and allowances are good, and a new field of scientific interest is opened up by the manifold problems which the circumstances of aviation produce. The physical and mental fitness for, and reaction to, the varied conditions under which the flying *personnel* perform their functions provide much scope for research. As promotion to the higher ranks of the Service is to be entirely by selection, and as a certain proportion of the higher ranks will be reserved for purely scientific as opposed to administrative appointments, it will be seen that there are excellent prospects for the young medical officer who exhibits ability and energy in scientific research, as well as for those who develop a talent for administration.

The establishment consists partly of permanent and partly of short-service officers. Short-service officers are admitted by direct entry for a period of two years, which may be extended to four years at the discretion of the Air Council on recommendation of the Director of Medical Services. Those who are not selected for permanent commissions pass into the Royal Air Force Medical Reserve at the expiration of their period of service on the active list, and will then receive gratuity to which their service entitled them. Short-service officers who are approved for permanent commissions, but for whom there are not vacancies in the Royal Air Force Medical Service, may under certain conditions transfer to the Royal Army Medical Corps, counting their time served in the Royal Air Force towards increments of pay and retired pay in the Royal Army Medical Corps. Officers selected for permanent commissions are granted leave for a period not exceeding nine months for post-graduate study in general medicine and surgery, tropical and preventive medicine, and other special subjects. Such leave is granted during the first six years of permanent service, and during such leave officers will remain on full pay and allowances.

New entrants into the Royal Air Force Medical Service are commissioned as Flying Officers (Medical), and are eligible for promotion to the rank of Flight-Lieutenant (Medical) after two years' service. Officers selected for permanent commissions will be promoted to the rank of Squadron Leader after ten years' total service. Accelerated promotion may be carried out, in a limited number of cases, of officers who show exceptional ability after the completion of eight years' service. Promotion to the rank of Wing Commander will be by selection at any period after sixteen years' total service, and to that of Group Captain by selection at any period after twenty-two years' service. There is no competitive examination on entry. A summary of the pay, allowances, etc., of this Service is given on p. 377.

